# SMX makes the cut in genome stability

**DOI:** 10.18632/oncotarget.22420

**Published:** 2017-11-13

**Authors:** Haley D.M. Wyatt, Stephen C. West

**Affiliations:** Haley D.M. Wyatt: Department of Biochemistry, University of Toronto, Toronto, Ontario, Canada

**Keywords:** genome stability, SLX1-SLX4, MUS81-EME1, XPF-ERCC1, macromolecular complex

The faithful duplication and preservation of our genetic material is essential for cell survival; however, DNA is susceptible to damage by extracellular and intracellular agents (e.g. ultraviolet radiation, reactive oxygen species). DNA double-strand breaks (DSBs) are thought to represent the most dangerous type of lesion, as the failure to repair a DSB can lead to loss of genetic information, chromosomal rearrangements, and cell death. Fortunately, cells contain sophisticated DNA repair pathways to counteract the deleterious effects of genotoxic agents. Mutations in DNA repair genes are linked to various diseases, including neurological defects, immunodeficiency, and cancer.

Gross chromosomal rearrangements, including deletions, duplications, and translocations, are a hallmark of cancer genomes; DSB formation is an obligate step that precedes these rearrangements. DNA sequence and structure also influence chromosome breakage and repair. For example, chromosome fragile sites (CFSs), which are highly repetitive loci that form DNA secondary structures, co-localize with genomic rearrangements in cancer cells [1]. In addition, DNA sequences that give rise to non-B-form DNA structures are frequently associated with translocation and deletion breakpoints in cancer genomes [2].

Structure-selective endonucleases cleave DNA secondary structures that arise during DNA replication and repair. In eukaryotes, the SLX1-SLX4, MUS81-EME1, and XPF-ERCC1 endonucleases are essential for genome stability; these enzymes remove branched DNA structures that would otherwise impede DNA replication and/or chromosome segregation. Examples of such structures include stalled replication forks and covalently linked, four-stranded recombination intermediates called Holliday junctions (HJs). Nevertheless, DNA cleavage opens the door for indiscriminate repair that can fuel genetic rearrangements and cancer development, emphasizing the importance of regulatory mechanisms to control endonuclease activity and prevent uncontrolled DNA cleavage.

Human SLX4 provides the scaffold for a tri-nuclease complex called SMX, comprised of SLX1-SLX4, MUS81-EME1, and XPF-ERCC1. Human SMX is the only known example of a tri-nuclease complex. As such, predictions about assembly and activation cannot be inferred from other systems. To this end, the SMX complex has been purified to address two fundamental questions: i) what are the DNA substrates of SMX?; and ii) how does the SLX4 scaffold co-ordinate three different nucleases for DNA cleavage? SMX was found to be a promiscuous endonuclease that cleaves a broad range of DNA secondary structures *in vitro*^3^. Remarkably, SLX4 co-ordinates the SLX1 and MUS81-EME1 nucleases during HJ resolution [3, 4] (Figure 1). The involvement of two active sites from different heterodimeric enzymes leads to a non-canonical mechanism of HJ resolution. It was also shown that SLX4 activates MUS81-EME1 to cleave structures that resemble stalled replication forks [3] (Figure 1). Activation involves relaxation of MUS81-EME1’s substrate specificity, which is regulated by a helix-hairpin-helix (HhH) domain in the MUS81 N-terminus (MUS81 N-HhH). Intriguingly, MUS81 N-HhH also mediates the interaction with SLX4 *via* a C-terminal SAP domain (SLX4 SAP) [5]. These observations led to the proposal that the MUS81 N-HhH domain acts as a self-inhibitory gate that regulates MUS81-EME1 nuclease activity [3]. Within the context of SMX, interaction of MUS81’s N-HhH domain with the SLX4 SAP domain may activate MUS81-EME1 by relaxation of substrate specificity. Nuclease activation ensures the removal of diverse DNA structures that would impede DNA replication and/or chromosome segregation.

**Figure 1:**
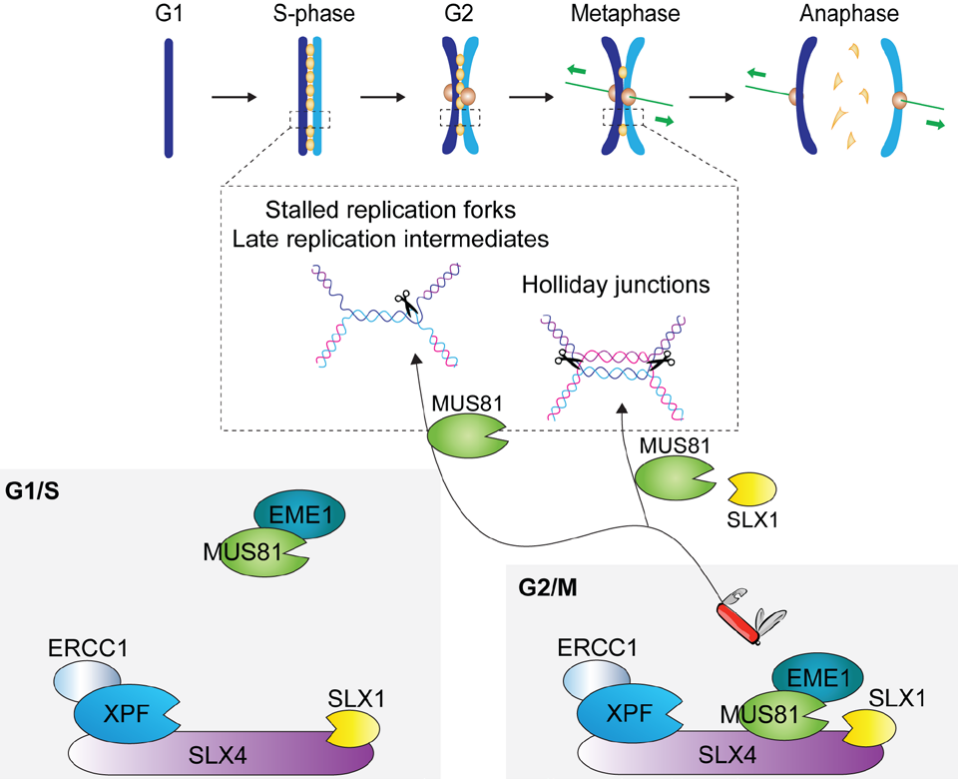
Cell cycle-regulated formation of the SMX tri-nuclease [3] The MUS81-EME1 subunit is recruited to a sub-complex comprised of SLX1-SLX4 and XPF-ERCC1 at a late phase of the cell-cycle, leading to the formation of SMX. Recombinant SMX cleaves a broad range of branched DNA structures (e.g. stalled replication forks, late replication intermediates, Holliday junctions) that would interfere with replication and/or chromosome segregation in the cell. The nucleases responsible for DNA cleavage depend on the DNA structure: MUS81-EME1 and SLX1 are required for coordinated Holliday junction resolution whereas MUS81-EME1 is activated to cleave replication-related structures. Figure is modified from the graphical abstract of Wyatt *et al.* [3].

The biochemical studies of SMX raise several interesting structural and biological questions about this macromolecular complex. For example, the finding that SLX4 alters the substrate specificity of MUS81-EME1 suggests that MUS81 undergoes extensive repositioning and/or conformational changes during SMX formation^3^. The structural details of these changes, and whether they accompany or precede substrate binding, are unknown. Another intriguing question is how the SLX1 and MUS81-EME1 active sites are coordinated during HJ cleavage [3, 4]. The involvement of two active sites from different heterodimeric enzymes is unique to SMX and represents a novel mechanism of HJ resolution in eukaryotes. Elucidating the structural details of these reactions will require high resolution models of the SMX complex in its apo- and DNA-bound states.

It is also intriguing to ask why cells have evolved this multi-nuclease complex. Most likely SMX provides an efficient tool to remove various DNA structures that would otherwise impede DNA replication and/or chromosome segregation. This proposal is supported by data showing that SMX-mediated cleavage of CFSs is necessary for genome stability [6]. Nevertheless, the human genome contains a plethora of branched and non-B DNA structures that represent potential SMX substrates, and unrestrained cleavage would lead to massive chromosome breakage. Cells must therefore contain mechanisms to regulate the activity of SMX. Indeed, SMX assembly is regulated by phosphorylation and constrained to a late phase of the cell cycle [3, 4, 7]. This temporal regulation is thought to prevent spurious chromosome cleavage whilst ensuring the efficient removal of HJs and branched DNA structures that arise at late replicating regions. A better understanding of the biological mechanisms that direct SMX activity to the appropriate time and place will reveal how cells prevent catastrophic chromosome cleavage.

## References

[1] Burrow AA (2009). BMC Genomics.

[2] Bacolla A (2016). Nucleic Acids Research.

[3] Wyatt HD (2017). Molecular Cell.

[4] Wyatt HD (2013). Molecular Cell.

[5] Nair N (2014). DNA Repair.

[6] Minocherhomji S (2015). Nature.

[7] Duda H (2017). Developmental Cell.

